# Podocyte Glucocorticoid Receptor Expression and Treatment Outcome in Idiopathic Nephrotic Syndrome

**DOI:** 10.1016/j.ekir.2025.04.034

**Published:** 2025-04-20

**Authors:** Bartholomeus T. van den Berge, Jitske Jansen, Jack F.M. Wetzels, Bart Smeets, Rutger J. Maas

**Affiliations:** 1 Department of Nephrology, Radboudumc, Nijmegen, The Netherlands; 2 Department of Pathology, Radboudumc, Nijmegen, The Netherlands; 3 Institute for experimental medicine and systems biology, Uniklinik RWTH Aachen, Aachen, Germany; 4 Department of Pediatric Nephrology, Radboudumc, Nijmegen, The Netherlands

**Keywords:** glucocorticoid receptor, idiopathic nephrotic syndrome, podocytes

## Introduction

Minimal change disease and focal segmental glomerulosclerosis are histologic manifestations of idiopathic nephrotic syndrome (iNS), which is considered a primary podocytopathy.^1^ Complete proteinuria remission is the aim of iNS treatment and is associated with long-term preserved kidney function. Partial remission is also associated with improved outcome; however, there remains a significant risk of relapses and progression.^2^ Most patients with iNS receive high-dose glucocorticoid (GC) treatment; however, the proteinuria response is highly variable and unpredictable in an individual patient.^3^, ^4^, ^5^ Increasing evidence suggests that beneficial effects of GC on proteinuria rely on direct podocyte stabilization via GC receptor (GR) signaling, in addition to suppression of putative immune-mediated factors.^6^ We hypothesized that reduced podocyte GR expression is associated with delayed treatment response to corticosteroids in patients with iNS.

## Results

To investigate this hypothesis, we measured podocyte-specific GR expression by immunofluorescence in biopsy material of 26 adult patients with iNS, 9 adult patients with membranous nephropathy (MN) as disease controls with nephrotic syndrome, and 9 adult controls who underwent (tumor-) nephrectomies. Detailed information about the study design, immunofluorescent staining, and formal analysis is provided in the Supplementary Methods and Supplementary Tables S1 and S2. In brief, kidney biopsies were stained for cell nuclei, podocyte nuclei, collagen type IV, and GR. In Supplementary Figure S1, we show representative images of control and iNS glomeruli, and the analysis of podocyte nuclear GR expression. Podocyte nuclear marker DACH-1 was used to identify podocyte nuclei, allowing podocyte-specific measurement of GR expression. Clinical characteristics, experimental measures, and disease outcome measures are described in Table 1. All patients with iNS received GC treatment with high-dose prednisone (monotherapy in 20 patients, and combination with additional immunosuppressive drugs in 6 patients). Median time interval between start of prednisone treatment and additional immunosuppression was 4 weeks (range: 0–5). The median follow-up time was 42 (interquartile range: 16–69) months. During follow-up, 100% and 73% of the patients reached partial remission and complete remission, respectively. Median time to first (partial) remission was 2.3 (interquartile range: 0.8–6.4) months. Patients with MN received various immunosuppressive regimens and the time between biopsy and initiation of immunosuppressive therapy varied widely. Therefore, no analyses were performed on the relationship between podocyte GR expression and time to remission in patients with MN.

**Table 1:** Clinical characteristics and experimental measures for controls and patients with iNS

Characteristics	Controls			Idiopathic nephrotic syndrome				Membranous nephropathy (disease controls)			
	*n*	Median	IQR	*n*	Median	IQR	*P*-value vs. control	*n*	Median	IQR	*P*-value vs. control
Age (yrs)	9	68	56–72	26	55	42–67	0.094	9	61	53–78	0.951
Sex (% male)	9	56%		26	69%		0.456	9	78%		0.317
Ethnicity (% Caucasian)	9	100%		26	100%		-	9	100%		-
Serum creatinine (μmol/l)	9	70	62–76	23	179	103–298	0.001	9	112	91–148	0.004
eGFR (ml/min per 1.73 m^2^)	9	94	81–105	24	37	16–80	0.001	9	61	46–75	0.105
Serum albumin (g/l)				22	18	13–25	-	9	19	16–28	-
UPCR (g/mmol)				26^a^	6.33	3.50–8.76	-	9	7.50	5.71–9.32	-
Podocyte specific nuclear GR expression (a.u.)	9	25.4	22.6–32.2	26	6.4	4.2–14.3	0.004	9	8.9	8.1–15.1	0.208
MCD/FSGS (*n*)				26	14/12		-				-
Immunosuppression							-				
Prednisone (%)				20	76.9%						
Prednisone + MMF (%)				4	15.4%						
Prednisone + tacrolimus (%)				2	7.7%						
Time to follow-up (months)				26	41.7	15.8–68.5	-				
Time to remission (months)				26	2.3		-				
MCD				14	1.3	0.8–6.4	0.053 vs. FSGS				
FSGS				12	5.6	0.7–4.5					
(% of total reaching PR/CR)				26	(100%/73%)	0.9–7.9					
					Low GR (< 6 a.u.)	High GR (≥ 6 a.u.)					-
Prednisone treatment response				26							-
Response ≤ 4 wks (yes/no)					1/12	7/6	0.030				
Response ≤ 8 wks (yes/no)					3/10	9/4	0.047				

a.u., arbitrary unit; CR, complete remission; eGFR, estimated glomerular filtration rate; FSGS, focal segmental glomerulosclerosis; GR, glucocorticoid receptor; iNS, idiopathic nephrotic syndrome; IQR, interquartile range; MCD, minimal change disease; MMF, mycophenolate mofetil; PR, partial remission; UPCR, urinary protein creatinine ratio.

*t* test or χ^2^-test were performed comparing controls versus patients with idiopathic nephrotic syndrome for continuous data and nominal data, respectively. Fisher exact test was performed comparing early and late responders, and low and high GR expression in patients with idiopathic nephrotic syndrome.

a All patients had nephrotic range proteinuria at time of presentation (≥ 3.5 g/10 mmol). Continuous data are expressed as median (IQR).

**Figure 1:**
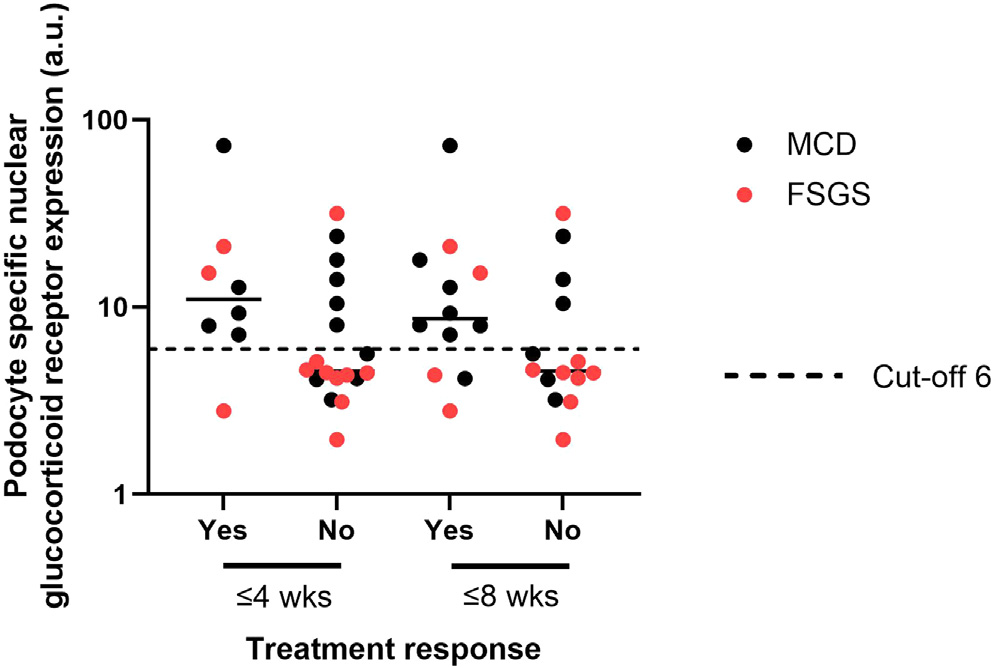
Mean podocyte-specific GR expression and treatment response in patients with iNS. Using the cut-off of 6 a.u., patients with high (≥ 6 a.u.) GR expression reached treatment response more often at 4 weeks (*P* = 0.030) and 8 weeks (0.047) compared with patient with low (< 6 a.u.) GR expression. a.u., arbitrary unit; FSGS, focal segmental glomerulosclerosis; GR, glucocorticoid receptor; iNS, idiopathic nephrotic syndrome; MCD, minimal change disease; wks, weeks.

Compared with nonproteinuric controls, podocyte GR expression was decreased in patients with iNS and MN, with mean expression lowest in iNS and with strong variation between patients (Supplementary Figure S2). In patients with iNS, podocyte GR expression did not correlate significantly with time to remission (*rho* = −0.23, *P* = 0.253), or proteinuria (*rho* = 0.08, *P* = 0.716). However, patients with iNS with high podocyte GR expression (≥ 6 a.u.) reached remissions at 4 (*P* = 0.030) and 8 (*P* = 0.047) weeks more often, compared with patients who had low GR expression (< 6 a.u.) (Table 1 and Figure 1). No statistically significant differences were observed between patients with minimal change disease and those with focal segmental glomerulosclerosis in terms of podocyte GR expression (*P* = 0.299) (Supplementary Figure S2) or time to remission (0.053) (Table 1). Similarly, patients who only reached partial remission had podocyte GR expression levels comparable to patients who reached complete remission.

## Discussion

Our data confirm previous observations of reduced glomerular GR expression in patients with iNS compared with nonproteinuric controls.^3^^,^^6^ Although our data suggest that reduced podocyte GR protein expression may be more pronounced in iNS compared with MN, further studies with larger cohorts are needed to confirm whether this finding truly differentiates iNS from other glomerular diseases. In addition, we showed that patients with iNS who had low podocyte GR expression (< 6 a.u.) on kidney biopsy were unlikely to reach GC-induced remissions at either 4 or 8 weeks. These observations suggest that GC responsiveness in patients with iNS may depend on podocyte GR signaling. Admittedly, there was an overlap in GR expression between early and late response in patients with iNS based on podocyte GR expression. We acknowledge that GC-responsiveness in patients with iNS is likely determined by several other factors, including medication compliance, pharmacokinetics, and immunologic factors.^7^ Several studies have reported a relationship in GC-responsiveness and GR expression in iNS kidney biopsy tissue, using immunohistochemistry and/or quantitative polymerase chain reaction to detect GR expression.^3^^,^^8^ We performed a reproducible quantitative immunofluorescence method and selectively measured GR expression in podocytes, whereas others measured the signal in unspecified glomerular cells. Contrary to Han *et al.*,^3^ we did not identify an overall correlation between time to GC response and GR expression in the kidney biopsy. Differences in methodology outlined above, and in patient populations may explain the discrepancy between studies. In conclusion, our findings supported our hypothesis that loss of podocyte GR expression is associated with delayed treatment response in patients with iNS. Given that many patients develop side effects during prolonged GC use, predicting GC responsiveness through the analysis of podocyte-specific markers in kidney biopsies may aid nephrologists’ clinical decision making. Our reproducible method for detection of podocyte-specific GR expression allows independent validation in other cohorts. Finally, our findings support research into podocyte-specific GR targeting in patients with iNS.^9^

## Disclosure

All the authors declared no competing interests.

## Supplementary Material

Supplementary File (PDF) **Supplementary Methods. Figure S1.** Podocyte glucocorticoid receptor expression. **Figure S2.** Mean podocyte nuclear glucocorticoid receptor expression in controls and patients with primary nephrotic syndrome. **Table S1.** Immunofluorescent staining overview of used chemicals, antibodies, working dilutions and antigen retrieval buffers. **Table S2.** Resource table.

## References

[1] Maas R.J., Deegens J.K., Smeets B., Moeller M.J., Wetzels J.F. (2016). Minimal change disease and idiopathic FSGS: manifestations of the same disease. Nat Rev Nephrol.

[2] Troyanov S., Wall C.A., Miller J.A., Scholey J.W., Cattran D.C., Toronto Glomerulonephritis Registry Group (2005). Toronto glomerulonephritis registry G. Focal and segmental glomerulosclerosis: definition and relevance of a partial remission. J Am Soc Nephrol.

[3] Han S.H., Park S.Y., Li J.J. (2008). Glomerular glucocorticoid receptor expression is reduced in late responders to steroids in adult-onset minimal change disease. Nephrol Dial Transplant.

[4] Rood I.M., Bavinck A., Lipska-Zietkiewicz B.S. (2022). Later response to corticosteroids in adults with primary focal segmental glomerular sclerosis is associated with favorable outcomes. Kidney Int Rep.

[5] Mirioglu S., Daniel-Fischer L., Berke I. (2024). Management of adult patients with podocytopathies: an update from the ERA Immunonephrology Working Group. Nephrol Dial Transplant.

[6] Broek M.V.D., Smeets B., Schreuder M.F., Jansen J. (2022). The podocyte as a direct target of glucocorticoids in nephrotic syndrome. Nephrol Dial Transplant.

[7] Schijvens A.M., Ter Heine R., de Wildt S.N., Schreuder M.F. (2019). Pharmacology and pharmacogenetics of prednisone and prednisolone in patients with nephrotic syndrome. Pediatr Nephrol.

[8] Gamal Y., Badawy A., Swelam S., Tawfeek M.S., Gad E.F. (2017). Glomerular glucocorticoid receptors expression and clinicopathological types of childhood nephrotic syndrome. Fetal Pediatr Pathol.

[9] Stamellou E., Agrawal S., Siegerist F. (2024). Inhibition of the glucocorticoid receptor attenuates proteinuric kidney diseases in multiple species. Nephrol Dial Transplant.

