# Supplementary material for: Podocyte Glucocorticoid Receptor Expression and Treatment Outcome in Idiopathic Nephrotic Syndrome
Source: Kidney Int Rep. 2025 Apr 20;10(7):2450–2. doi: 10.1016/j.ekir.2025.04.034 (PMC12266189; doi:10.1016/j.ekir.2025.04.034)
Supplement: Supplementary File (PDF) — Supplementary Methods. Figure S1. Podocyte glucocorticoid receptor expression. Figure S2. Mean podocyte nuclear glucocorticoid receptor expression in controls and patients with primary nephrotic syndrome. Table S1. Immunofluorescent staining overview of used chemicals, antibodies, working dilutions and antigen retrieval buffers. Table S2. Resource table. [file mmc1.pdf]

Podocyte glucocorticoid receptor expression and treatment outcome in idiopathic nephrotic syndrome

Bartholomeus T van den Berge[1,2], Jitske Jansen[3,4], Jack FM Wetzels[1], Bart Smeets[2], Rutger J Maas[1¥]

[1]Department of Nephrology, Radboudumc, Nijmegen, The Netherlands. [2]Department of Pathology, Radboudumc, Nijmegen, The Netherlands. [3]Institute for experimental medicine and systems biology, Uniklinik RWTH Aachen, Aachen, Germany. [4]Department of Pediatric Nephrology, Radboudumc, Nijmegen, The Netherlands.

[¥]Corresponding author

Rutger.Maas@radboudumc.nl

Geert Grooteplein Zuid 10, 6525 GR Nijmegen, The Netherlands

Supplementary Material

Page 2: Supplementary figure S1. "Podocyte glucocorticoid receptor expression"

Page 3: Supplementary figure S2. "Mean podocyte nuclear GR expression in controls and patients with primary nephrotic syndrome."

Page 4: Supplementary Methods.

Page 7: Supplementary table S1. "Immunofluorescent staining overview of used chemicals, antibodies, working dilutions and antigen retrieval buffers."

Page 8: Supplementary table S2: "Resource table."

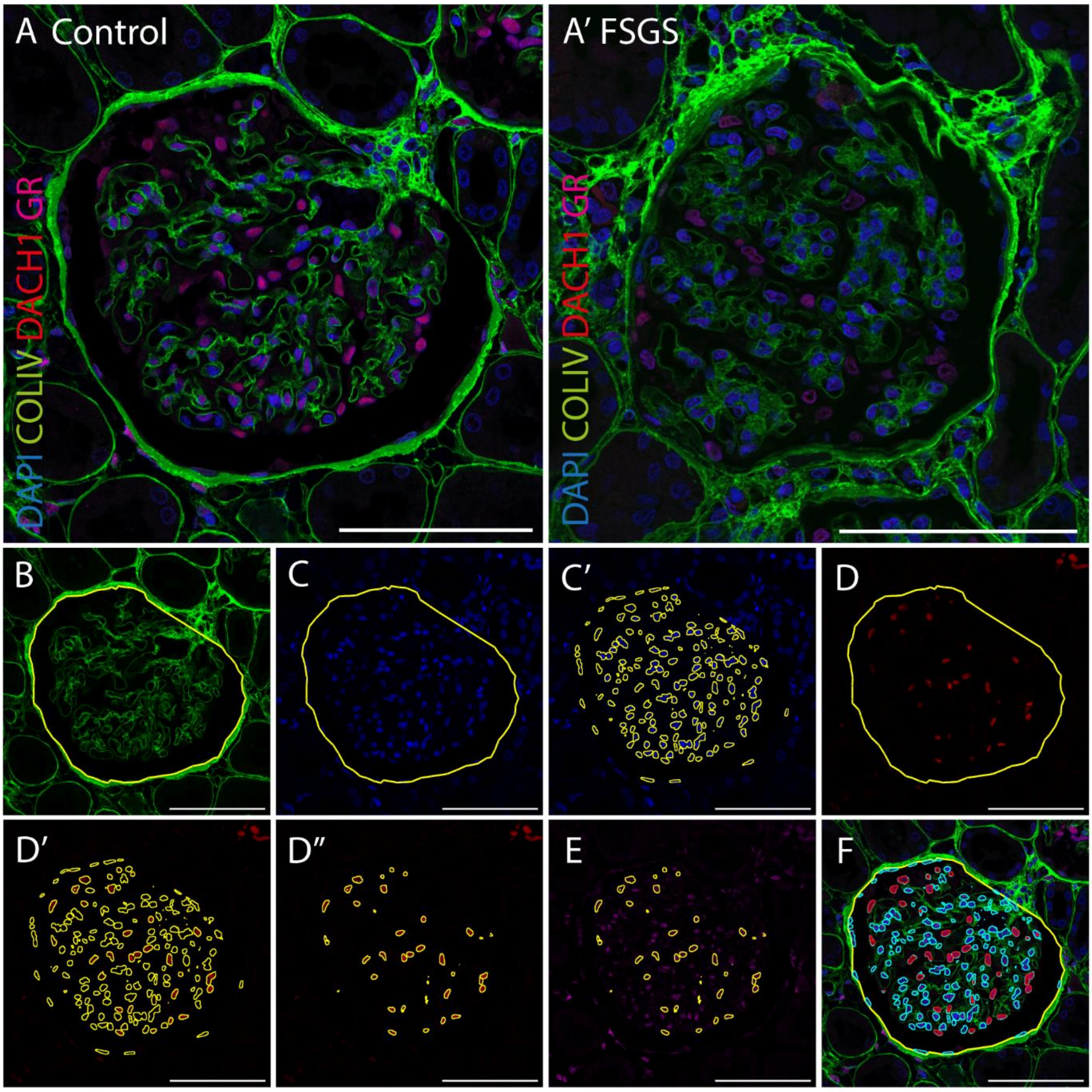

Supplementary figure S1. Podocyte glucocorticoid receptor expression. (A-A') Representative overviews of a healthy control glomerulus (A), and glomerulus of a patient with FSGS (A'). Biopsies were stained for DAPI (blue), collagen type IV (green), dachshund family transcription factor 1 (DACH1, red) and glucocorticoid receptor (GR, magenta). (B) Glomerular annotation (yellow) using COLIV (green). (C-C') Glomerular nuclei (DAPI) annotation (C') using the COLIV glomerular annotation (C). (D-D") DAPI$^+$/DACH1$^+$ podocyte nuclei annotation (D") using the COLIV glomerular annotation (D) and then thresholded glomerular DAPI (D') as selections. (E) Podocyte-specific nuclear GR annotation using the DAPI$^+$/DACH1$^+$ glomerular annotation (D") as selection. (F) Overview of all glomerular (yellow), glomerular nuclei (cyan) and podocyte (red) annotations. FSGS: Focal segmental glomerulosclerosis. Scale bars represent 100 µm.

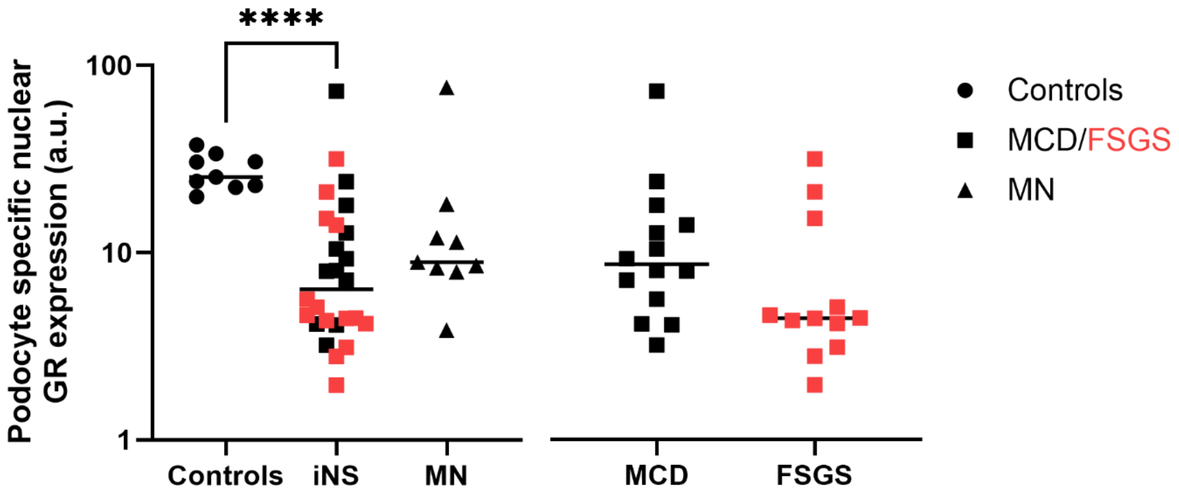

Supplementary figure S2. Mean podocyte nuclear GR expression in controls and patients with primary nephrotic syndrome. Patients with iNS had statistically significant lower mean podocyte-specific nuclear GR expression when compared with controls. Podocyte-specific nuclear GR expression did not differ significantly between MCD and FSGS patients (p=0.299). iNS: idiopathic nephrotic syndrome; MCD: minimal change disease; FSGS: focal segmental glomerulosclerosis. MN: Membranous nephropathy. **** P≤0.0001 (Student's t-test).

Supplementary Methods

Study design

A retrospective study was performed in adult patients with idiopathic nephrotic syndrome (iNS) and membranous nephropathy (MN), who were referred to Radboudumc (a university hospital) between 2017-2023. INS and MN patients were identified from our clinical registry and the pathology database. Inclusion criteria included nephrotic syndrome (proteinuria ≥3.5 g/10 mmol or 3.5 g/24h and hypo-albuminemia) and histopathological diagnosis of MN, MCD or FSGS. Permission for the use of archived material and patient data was obtained by the local ethical commission for human medical research of the Radboud University Medical Center, Nijmegen, The Netherlands (approval number: **2018-4086**). Kidney biopsy tissue from patients with iNS, MN and controls without evidence of kidney disease was used. Control kidney tissue was obtained from the unaffected part of (tumor-)nephrectomies and PAS stainings confirmed normal morphology in those sections. Controls did not have proteinuria and had no history of kidney disease before (tumor-)nephrectomy. For definitions of clinical outcomes we followed the KDIGO 2021 guidelines (Kidney Int, 2021). In short, patients were considered in complete remission (CR) if proteinuria was ≤0.3 g/10 mmol creatinine. Patients were considered in partial remission if proteinuria was between ≥0.3 g/10 mmol and ≤3.5 g/10 mmol, and a decrease was observed of ≥50% from onset. Estimated glomerular filtration rate (eGFR) at time of biopsy was calculated using the Chronic Kidney Disease Epidemiology Collaboration (CKD-EPI) equation.” All data were coded. Immunofluorescent stainings were performed on paraffin embedded kidney biopsy material of patients with iNS, MN and healthy controls.

Immunofluorescent staining

Paraffin embedded kidney biopsy material of patients with iNS, MN and controls was deparaffinized using a series of xylol (2x) and 100% (v/v) ethanol (3x). Tissue slides were rinsed in demineralized water once after which antigen retrieval was performed by boiling tissue slides in their respective buffers for 3 min at 600 W and another 60 min at 180 W. Tissue slides were cooled down on ice for 20 min and blocked with 10% donkey blocking serum in PBS (DBS, Gibco) for 10 min. Primary (1:100) and secondary (1:200) antibodies were diluted in PBS containing 1% (v/v) bovine serum albumin (BSA, Sigma). Primary antibodies were incubated overnight at 4°C and secondary antibodies at room temperature for 2 hours. Between and after antibody incubations, tissue slides were washed for 10 min in their respective buffers. Tissue slides were mounted with Fluormount-G® (Southern Biotech, SanBio) and covered with glass cover slips. Images were captured using a Zeiss LSM 880 confocal microscope. All relevant information of respective buffer and antibody dilutions used are listed in Supplementary table S1.

Podocyte specific nuclear GR expression

Podocyte specific nuclear GR expression was measured (as a marker for translocated (active) GR) using fluorescent images obtained of DAPI, COLIV, DACH1 and NR3C1 (GR). First, glomerular outline was annotated using COLIV and used a selection for glomerular nuclei. Secondly, glomerular nuclei were used to select DACH1^{+} nuclei. Thirdly, DAPI^{+}/DACH1^{+} podocyte nuclei were selected and used as an overlay on the NR3C1 (GR) image. GR expression was measured in all podocyte nuclei individually and mean podocyte specific nuclear GR expression per glomerulus was calculated. Lastly, mean podocyte specific nuclear GR expression was calculated (Supplementary figure 1). For this analysis, custom-made

macros were developed in Fiji and the script was deposited on Zenodo (Zenodo 10.5281/zenodo.13143241) (Supplementary table S2).

Supplementary table S1. Immunofluorescent staining overview of used chemicals, antibodies, working dilutions and antigen retrieval buffers.

**Primary antibody or chemical**	**Working dilution**	**Secondary antibody**	**Working dilution**	**Antigen retrieval buffer**
Podocyte specific nuclear GR				
DAPI (D1306, Invitrogen)			1:1000	Citrate
Goat Anti-Type IV Collagen-UNLB (1340-01, Southern Biotech)	1:100	Donkey anti-Goat Alexa Fluor™ 488 (A-11055, Thermo Fisher)	1:200	
Dachshund 1 (MABS1805M, Sigma-Aldrich)	1:100	Donkey anti-mouse Alexa Fluor™ 568 (A10037, Thermo Fisher)	1:200	
Anti-NR3C1 (HPA004248, Sigma-Aldrich)	1:100	Donkey anti-rabbit Alexa Fluor™ 647 (A-31573, Thermo Fisher)	1:200	

Supplementary table S2. Resource table.

**Deposited Data**		
Scripts and codes for data analysis	This paper; deposited on Zenodo	10.5281/zenodo.13143241
**Software and Algorithms**		
ImageJ version Fiji 1.51n	National Institutes of Health, USA	https://imagej.nih.gov/ij
Adobe Illustrator CC 2021	Adobe Systems Inc.	RRID:SCR_010279
Adobe Photoshop CC 2021	Adobe Systems Inc.	RRID:SCR_014199
GraphPad Prism version 9.5	GraphPad Software Inc.	RRID:SCR_002798
SPSS version 29	IBM SPSS statistics 29	RRID:SCR_016479
